# Medicinal Aid in Parturition

**Published:** 1889-03

**Authors:** B. L. B. Baylies

**Affiliations:** Brooklyn


### MEDICINAL AID IN PARTURITION.

B. L. B. Baylies, M. D., Brooklyn.

It would be superfluous and ridiculous to attempt the aid of a wisely constructed and adapted organ in the normal and healthy performance of its function. "Meddlesome midwifery is bad." Normal and healthy parturition requires no assistance; there should be no interference, digital or instrumental, with labor, which can possibly be avoided. By reason of the danger of disturbing the harmonious nervous and muscular action of the parturient organs, it is best, if certainly ascertainable, to determine the position of the child by palpation through the abdominal walls, and frequently better that the obstetrician, leaving the patient in charge of a well-chosen nurse, should only be within hearing distance, interpreting the utterance or cries of his patient, as significant of the stage of the process; ready to give aid, comfort, or encouragement when required. The physical system is, however, usually disturbed by the pregnant state, or the parturient function; its latent dyscrasias so awakened into activity, that homœopathic medication will often operate most *essentially*, to defeat the rheumatic, psoric, or other antagonist, and prepare the way for easy delivery.

How often has the suitable remedy, administered during gestation or parturition, imparted health, promoted the normal development of the fœtus, aided labor, and sometimes prevented death of mother or child. I will present for illustration the operation of some of these remedies:

The woman is anxious, restless, fearful of death; predicts the time of death; face hot, flushed, with frightened expression; acute fever, pulse full, strong, and hard; giddy while sitting, pains of labor distressing; vagina hot and dry; violent labor-pains in rapid succession, especially with a large child; shrieking; red, sweaty face (Hering). A single dose of Aconite in such a case relieves the patient's terror, and composes her to assist the regular efforts of labor.

When the labor is powerless or irregular, as a consequence of excessive exertion or fatigue, with sensation of weariness; painful sensitiveness of the body to pressure; soreness apparently resulting from abnormal sensibility of the neck of the uterus to the pressure of the child's head; false pains will be removed, or the feeble action quickly rendered efficient by Arnica.

Belladonna for "violent pressing toward the genitals as if everything would fall out there;" or labor-pains deficient, cease, only periodical, slightly pressive on the sacrum (I quote Hering's *Guiding Symptoms*); amniotic fluid gone, yet the os seems spasmodically contracted; first delivery, muscles rigid. For two primiparæ, when the head, being in the inferior strait, pains had suddenly ceased, a dose dry of Bell.$^{97m}$ (Fincke) induced powerful labor immediately, and the birth so instantaneously followed as to alarm me, with apprehension of rupture of the perinæum.

Chamomilla: Drawing pain in the back, and stitches in the back; sensation as if the lumbar region would be broken, with dragging and drawing pains extending from the region of the liver, over the abdomen, and deep into the pelvis, when lying in bed; dragging toward the ureters like labor-pains, with frequent urging to urinate; drawing from the sacral region forward, griping and pinching in the uterus, followed by discharge of large clots of blood (placenta previa?); flushed face, irascible and impatient temper; convulsions in the back, with throwing backward of the head, and stiffness of the body, as in tetanus (Allen's *Encyclopædia*, Jahr's *Symptomatology*). (Puerperal convulsions of tetanic character.) "Threatened abortion, with discharge of dark blood, great restlessness and agony," labor-pains press upward; she is hot, thirsty, and inclined to scold, labor-pains spasmodic and distressing, tearing pains down the legs; she is over-sensitive to the pain and wishes to get away from herself; hour-glass contractions; rigidity of the os; after-pains; powerful convulsions (Hering, *Guiding Symptoms*).

Pulsatilla: Drawing, pressive pain extending toward the uterus, with qualmishness; contractive pain in the left side of the uterus, like labor-pain, obliging her to bend double; violent cutting pain low down in the abdomen, with a sensation as if a stool would occur (a common and annoying symptom in labor); cutting, dragging pains in the hypogastrium, extending around to the loins, and making her feel faint (verified) (Allen's *Encyclopædia*). In threatened abortion, when the flow ceases, and then returns with double force, ceases again, and so on, promotes expulsion of moles (Hering's *Condensed*). When labor-pains are irregular, deficient, or sluggish; excite suffocation, or faint spells; for retained placenta from inaction, or hour-glass contraction of the womb. It has also in numerous cases seemed to correct mal-presentation.

Nux vomica: Extreme pains, apparently constrictive, in the first stage of labor, impeding dilatation of the os; in the last stage, the expulsion of the placenta; crampy pains with flatulence, bruised feeling of the abdominal wall or intestines, with frequent desire to defecate and urinate; each pain attended by sudden sharp cramps in the calves of the legs; the extremities cool; sensibility to currents of cool air. A dose of Nux v.$^{25m}$ has often promptly removed these symptoms. I have given Actea racemosa for distressing and protracted pains in the region of the cervix, during the first stage of labor; with the apparent effect to arrest pain and postpone labor. Hering's *Guiding Symptoms* gives for it these indications: "Spasms of the broad ligaments; ovarian pains shoot up to the side; darting pains in the uterine region, from side to side; pains worse from motion; bearing down in the uterine region and small of the back; false labor-pains; sharp pains across the abdomen; fainting fits or cramps, with severe, tedious, or spasmodic labor-pains; cardiac neuralgia in parturition; in labor, rigidity of the os uteri; crampy and stitching pains in the limbs; limbs heavy and torpid. Given during the last month of gestation, it shortens labor." Stitching pains and torpidity of the limbs; and shooting transversely in the uterine region, distinguish it from Caulophyllum. The symptoms indicating Caulophyllum are: Sensation as if the uterus were congested, with fullness and tightness in the hypogastrium; prickings as from needles in the cervix, spasmodic rigidity of the os, delaying labor; accompanied by tremulous weakness; severe spasmodic bearing-down pains in the back and loins; labor-pains short, irregular, and spasmodic (Hering). Successful experience with Caulophyllum in dysmenorrhœa, with severe intermittent spasmodic drawing pains in the region of the broad ligaments, and nervous weakness, suggested to me their resemblance to symptoms sometimes occurring in indolent labor. I formerly gave it much in the 2d centesimal potency to promote expulsion of the placenta, but, with more discriminating selection, now rarely use it.

Lycopodium: During the labor-pains she must keep in constant motion, with weeping. Labor-pains run upward (Hering). After-pains, with sticking in the right or left iliac region, dragging toward the inguinal region; urging to urinate, but inability to do so, with constant bearing-down feelings; retention of urine.

Calcarea carb.: Jerk-like tearing down the sides of the abdomen; griping and cutting in the hypogastrium; dragging in the groin; burning, sore pains in the genitals. A case of excessive menstrual flow, twice in succession, occasioning the expulsion of a small fœtus; with a sort of labor-pain; cutting and bearing down in the hypogastrium, and violent desire for stool (Hahnemann). Hering recommends it in threatened miscarriage in those who generally have profuse menses, or are subject to hemorrhage. A fleshy and lymphatic, clumsily-moving woman, subject to profuse general perspiration, and troubled also, during pregnancy, with anasarca; who formerly had several still-births at term, improved in health and bore a healthy living child after taking Calcarea at intervals during gestation. A colored woman whose previous children had died within a few weeks after birth from profuse hemorrhages per anum, and who was herself subject to exhausting hemorrhoidal flux, took Calcarea c.$^{25m}$ (Fincke) during pregnancy and gave birth to a healthy child.

Secale cornutum : During labor, prolonged bearing down and forcing pains in the uterus; pains irregular, feeble, ceasing. Everything seems loose and open; retained placenta, with strong bearing down, or with relaxed feeling of the parts; after-pains long and painful (Hering). Aversion to heat and wrapping up. Sensation of remarkable coldness in the abdomen and back; horripilation of the abdomen, back, and limbs; diarrhœa, discharges from the vagina almost black, fluid, and very fetid. Puerperal metritis; miscarriage followed by tearing pains in the extremities. Tetanic rigidity. Epileptiform convulsions. The symptoms indicate its use in metritis, with septicemia.

Hyoscyamus: According to Dr. Hering, spasms during parturition, with nervous irritability. Every ten or fifteen minutes an attack of twitching of the limbs, and of the muscles of the face; unconsciousness; puerperal spasms; shrieks, anguish; chest oppressed, unconscious; after miscarriage or labor, hemorrhage of bright-red blood, flowing steadily; commences with spasms, single shocks; twitching and startings; with every start, more blood comes. Puerperal fever; no will to make water in child-bed. Watery, painless diarrhœa of lying-in women. Total suppression of milk or lochia (Hering's *Guiding Symptoms*). I have unfailingly used it to relieve retention of urine in child-bed.

Gelsemium: Great lassitude, complete muscular relaxation with motor paralysis; dullness of mind, relieved by profuse emissions of urine. Severe, sharp labor-like pains in the uterine region, extending to the back and hips. "Labor gone; os widely dilated, complete atony; drowsy. Albumenuria. Labor delayed by rigid os, or when pains go from before backward; the uterus seems to go upward; sensation like a wave from the uterus to the throat, ending with a choking feeling. This seems to impede labor" (Hering's *Condensed*).

Each of these, like many other drugs in a potentized form, is a beneficent agent, the characteristic features of which must be recognized, and its similitude perceived, to determine the sphere of its ministry.—*Bureau of Obstetrics, I. H. A.*.

